# Application of Chitosan-Based Coatings Incorporating Sea Fennel Essential Oil and Rosemary Extract on the Chemical and Microbiological Characteristics of Sardine (*Sardina pilchardus*) and Gilthead Seabream (*Sparus aurata*)

**DOI:** 10.3390/foods15203578

**Published:** 2026-10-09

**Authors:** Tibor Janči, Tea Sokač Cvetnić, Iva Čanak, Petra Babić, Emili Vidan, Nasreddine Benbettaieb, Frédéric Debeaufort, Mia Kurek

**Affiliations:** 1 Faculty of Food Technology and Biotechnology, University of Zagreb, Pierotti Street 6, 10000 Zagreb, Croatia; tjanci@pbf.hr (T.J.); tsokac@pbf.hr (T.S.C.); iva.canak@pbf.unizg.hr (I.Č.); ppisonic@pbf.hr (P.B.); evidan@pbf.hr (E.V.); 2 BioEngineering Department, IUT-Dijon, University Burgundy Europe, 7 Blvd. Docteur Petitjean, BP17867, 21078 Dijon CEDEX, France; nasreddine.benbettaieb@ube.fr (N.B.); frederic.debeaufort@ube.fr (F.D.); 3 PCAV Team, Joint Unit UMR PAM, Food Processing and Microbiology, INRAe, Institut AgroDijon, University Burgundy Europe, 1 Esplanade Erasme, 21000 Dijon, France

**Keywords:** sardines, gilthead seabream, edible coating, chitosan, gelatin, sea fennel, rosemary

## Abstract

Edible coatings have attracted increasing interest in the food industry owing to their biodegradability, non-toxicity, and potential to extend the shelf life of refrigerated fish by reducing lipid oxidation and microbial spoilage. This study evaluated the species-dependent effectiveness of bioactive edible coatings based on chitosan or chitosan–gelatin, enriched with sea fennel (*Crithmum maritimum* L.) essential oil and aqueous rosemary (*Rosmarinus officinalis*) extract. Two Adriatic fish species with contrasting biochemical characteristics, sardine (lipid-rich, pelagic) and gilthead seabream (lean, demersal), were coated and stored at 4 °C. Quality changes were monitored by sensory evaluation, thiobarbituric acid reactive substances (TBARS), proteolytic index, total volatile basic nitrogen (TVB-N), and microbiological analyses. Lipid oxidation, proteolysis, and TVB-N increased during storage, particularly in sardine. Chitosan-based coatings reduced proteolytic degradation more effectively than chitosan–gelatin coatings, although neither formulation significantly limited lipid oxidation in sardine. In contrast, the coatings were more effective in preserving the quality of gilthead seabream. These results demonstrate that the performance of edible coatings strongly depends on fish species, reflecting differences in biochemical composition and dominant spoilage pathways. The findings also highlight the importance of coating formulation, particularly the use of monopolymer versus blended systems, for developing effective preservation strategies for different fish products.

## 1. Introduction

Growing global concerns about food waste, environmental pollution and the sustainability of food systems are resulting in the development of innovative preservation and packaging strategies, especially for perishable products such as fish [1,2]. Thus, preserving perishable foods such as fish is no longer just a technological challenge, but a key issue that focuses on the simultaneous reduction in food waste, environmental sustainability and regulatory transformation. Seafood is among the most vulnerable food categories due to its rapid post-mortem biochemical degradation and microbial spoilage, which significantly contributes to food losses in the supply chain [1]. At the same time, conventional fish packaging systems, which are often based on multi-layer, non-degradable plastics, pose serious environmental challenges, as they are difficult to recycle and contribute to long-term pollution. Also, conventional preservation strategies rely heavily on cold chains that require significant resources and multilayer plastics, which are difficult to recycle and increasingly incompatible with evolving environmental policies and circular economy goals. This dual pressure includes reducing food and packaging waste, and requires a paradigm shift in the way preservation systems are designed and finally applied [3]. Rather than viewing packaging and preservation techniques as independent components, recent research trends are moving towards integrated systems in which part of the preservation function is transferred directly to the food surface [4]. In this framework, edible coatings serve as protective barriers and functional interfaces that can modulate physicochemical and microbiological processes during storage and potentially enable the use of simpler, less multi-layered external packaging. This combined approach may reduce reliance on complex multilayer plastics while maintaining product quality and safety [5,6].

Within this context, edible coatings have emerged not only as preservation tools but also as functional components of sustainable packaging. They act as semi-permeable barriers that limit the transfer of oxygen, moisture, and other environmental impacts to the fish surface. Consequently, oxidative processes and other deterioration reactions can be slowed. In addition, coatings can serve as carriers of bioactive compounds, such as antioxidants and antimicrobial agents. Their delivery at the food surface, where spoilage reactions are most pronounced, can help to delay oxidative and microbial deterioration while simultaneously enhancing the functional value of fish products [7,8,9].

The development of such active edible coatings must, however, be within existing regulatory requirements. In the European Union, the use of active substances in food contact materials is regulated under Commission Regulation (EC) No 450/2009 [10], which governs active and intelligent packaging systems. In addition, incorporated compounds must comply with food safety legislation, including Regulation (EC) No 1333/2008 [11] on food additives. Also, it is worth mentioning the regulations that are related to the plastic and recycled plastic materials for food products [12,13]. These regulations impose specific restrictions regarding the authorization, migration, and labelling of active substances, particularly when they are intended to exert technological effects on the food product, such as extending shelf-life. Therefore, the selection of bioactive compounds and coating matrices must consider not only functional performance but also regulatory compliance and consumer acceptance.

Among biopolymers, chitosan and gelatin have attracted considerable attention from both industry and researchers due to their film-forming capacity, biocompatibility, antimicrobial, and/or antioxidative properties [7,14,15,16]. However, the performance of edible coatings depends not only on the choice of polymer but also on the structural design of the coating system. Blending polymers such as chitosan and gelatin could potentially combine their individual advantages, although synergistic or antagonistic effects may occur and require systematic evaluation. Blending chitosan to gelatin forms a polyelectrolyte complex [14,15] with excellent physical and mechanical properties [14,17]. These films effectively protect food products, facilitate transportation, ensure hygiene, and extend the shelf life of perishable items, particularly those sensitive to stress, air, light, and temperature fluctuations [14]. The application of a chitosan–gelatin film showed potential as a food preservative and prolonged the shelf life of different food products, for example, of chicken meat [9], rainbow trout [18] and fish patties [19]. However, systematic comparisons between monopolymer and blended coating systems, particularly regarding their interaction with incorporated bioactive compounds, remain limited.

The incorporation of plant-derived bioactives further adds complexity and innovation potential to coating systems. Rosemary (*Rosmarinus officinalis*) is a well-established source of antioxidant and antimicrobial compounds, widely used in food applications [20]. A previous study showed that rosemary aqueous extract exhibits higher antioxidant and antimicrobial activity compared to the aqueous extract of sea fennel, supporting its application as an effective natural preservative [21]. In addition, rosemary essential oil has already been extensively investigated, limiting the novelty of further studies [22,23]. In contrast, sea fennel (*Crithmum maritimum* L.), a halophytic plant adapted to coastal environments, remains relatively underutilized despite its promising composition of bioactive molecules [21,24,25]. Particularly, its essential oil has not been extensively investigated in seafood preservation, representing an opportunity for the valorization of this species in line with biodiversity utilization and the exploration of novel natural ingredients. When incorporated into a chitosan matrix, these bioactive compounds may exhibit enhanced effectiveness, as chitosan itself possesses film-forming, oxygen-barrier, and antimicrobial properties. Furthermore, the polymer network can protect volatile compounds from rapid loss and enable their gradual release at the fish surface, potentially extending and specifically targeting their biological activity during storage. Thus, the combination of chitosan with rosemary extract and sea fennel essential oil is expected to provide complementary barrier, antioxidant, and antimicrobial effects and thereby contribute to improved preservation of fish quality. Therefore, this study aimed to both study the proven efficacy of rosemary aqueous extract and to provide an insight into the not yet well investigated potential of sea fennel essential oil for seafood applications. In our earlier studies, the focus was primarily on the characterization of plant-derived bioactive extracts and essential oils [21], including the evaluation of their antioxidant and antimicrobial properties, as well as their direct application to fish products without the use of a supporting carrier matrix. In contrast, the present work advances this research by incorporating these bioactives into chitosan and chitosan–gelatin edible coating matrices and evaluating whether the polymeric system itself can provide an additional protective effect through barrier properties, controlled release of active compounds, and enhanced preservation efficiency during chilled storage [26].

To the best of our knowledge no study has comprehensively evaluated the effect of chitosan–gelatin-based coatings enriched with plant-derived bioactive compounds, such as rosemary aqueous extract and sea fennel essential oil, on the quality of different fish species like sardines and gilthead seabream that are characterized by distinct spoilage mechanisms. By combining coating performance across different polymer matrices, bioactive systems, and fish species, this study aims to provide a more practical approach and comparative evaluation for developing sustainable preservation strategies for fresh fish.

The effectiveness of edible coatings is strongly influenced by the intrinsic properties of the food matrix. Fish species differ markedly in lipid content, muscle composition, and dominant spoilage pathways. Small pelagic species such as sardine have a high lipid content rich in long-chain n-3 polyunsaturated fatty acids (PUFA), particularly eicosapentaenoic (EPA) and docosahexaenoic acid (DHA), which makes them highly susceptible to lipid oxidation, whereas lean species such as gilthead seabream spoil predominantly through microbial and proteolytic processes. The fatty acid profiles of the fish used in this study are given in Table S1. Post-mortem deterioration of fish results from endogenous enzymatic autolysis, microbial growth and lipid oxidation. Endogenous enzymes and spoilage microorganisms break down proteins and non-protein nitrogenous compounds, leading to the accumulation of volatile basic compounds such as ammonia, trimethylamine and dimethylamine, measured as TVB-N [27,28,29]. At the same time, the oxidation of PUFA produces secondary oxidation products, including malondialdehyde, determined by TBARS analysis. These changes are accompanied by changes in pH, accumulation of proteolytic products and loss of sensory acceptability [27].

Although chitosan-based coatings and rosemary-derived bioactives have been widely studied, three aspects remain insufficiently explored: (i) the use of sea fennel essential oil as an active agent in edible coatings for fish; (ii) whether a monopolymer (chitosan) or a blended (chitosan–gelatin) coating is the more effective carrier of the same bioactive compounds when applied to fish; and (iii) whether the same coating systems perform similarly on fish species with contrasting composition and spoilage pathways. In our previous work, the bioactive profiles of rosemary and sea fennel extracts were characterized [21], and their aqueous extracts were applied directly to sardine and gilthead seabream without a carrier matrix [26]. Evaluating coating performance across such different systems is needed for a more comprehensive understanding of their functionality and applicability [6]. The present study extends this work by incorporating these bioactives into chitosan and chitosan–gelatin coatings. Therefore, the objective of this study was to evaluate the effect of chitosan and chitosan–gelatin coatings enriched with rosemary aqueous extract or sea fennel essential oil on the sensory, chemical and microbiological quality of sardine and gilthead seabream during storage at 4 °C. The study focused on the performance of the coatings on the fish product; characterization of the physicochemical and mechanical properties of the coatings themselves was beyond its scope. To the best of our knowledge, this is one of the first study to (i) apply sea fennel essential oil in chitosan-based coatings for fresh fish, (ii) directly compare a monopolymer and a blended coating as carriers of the same bioactives on the same fish species, and (iii) show that microbial inhibition by an active coating does not necessarily extend the sensory shelf life of a lipid-rich pelagic fish, while the same coatings are more effective on a lean species.

## 2. Materials and Methods

### 2.1. Materials

Sardines (*Sardina pilchardus*) (16.52 ± 2.00 g, and 12.52 ± 0.57 cm) were purchased at the fish market (Tržnice Zagreb, Zagreb, Croatia), and gilthead seabream (*Sparus aurata*) (125.74 ± 14.50 g, and 19.30 ± 2.45 cm) fillets were obtained from a local aquaculture producer Cromaris d.d. (Zadar, Croatia). Seabream fillets were machine filleted, packed in ice and transported to the laboratory within 12 h from harvest. The active extract was prepared from rosemary (*Rosmarinus officinalis* L.), purchased from Suban Ltd. (Strmec Samoborski, Croatia), and a sea fennel essential oil was purchased from OPG Grgo Lučić (Nerežišće, Island Hvar, Croatia). Extracts were always freshly prepared. Films were made from the natural biopolymer chitosan (chitosan type 652, molecular weight 165 kDa, degree of deacetylation above 85%, France Chitin, Marseille, France) and gelatin derived from fish (Louis Frnacois, Croissy Beaubourg, France).

Butylhydroxytoulene (BHT, 99.8%), trichloroacetic acid (TCA), 2-thiobarbituric acid (TBA, ≥98%), and cadmium chloride hemipentahydrate were purchased from Acros Organics (Geel, Belgium). 1,1,3,3-tetrametoxypropane (TMP, 99%) and L-leucine were purchased from Sigma Aldrich (Steinheim, Germany). Boric acid was purchased from Kemika d.d. (Zagreb, Croatia), and ethanol was purchased from Gram mol Ltd. (Zagreb, Croatia). Hydrochloric acid was supplied from Carlo Erba Reagents S.A.S. (Peypin, France). Glacial acetic acid (1% *v*/*v*) was purchased from J.T. Baker (Schwerte, Germany).

### 2.2. Methods

#### 2.2.1. Preparation of Rosemary Extract

Firstly, dry plant material was crushed in the grinder (HR2860/55, Philips, Amsterdam, The Netherlands), and then mixed with distilled water used as the solvent for the extraction at a ratio of 0.05 g plant/mL. The extraction was carried out in a round-bottomed flask placed in a microwave reactor (Start S Microwave Labstation for Synthesis, Milestone, Sorisole (Bergamo) Italy). The extraction was conducted for 10 min at a power of 500 W and at a temperature of 70 °C. After extraction, the sample was filtered through 100% cellulose filter paper (Whatman n◦1, Ø = 110 mm) to separate the aqueous extract from the solid phase. The filtered extract was stored at 4 °C until the coating preparation.

#### 2.2.2. Preparation of Coating Solutions

The individual 1.5% (*w*/*v*) coating solutions were prepared by dissolving chitosan (CS) in 1% (*v*/*v*) acetic acid or gelatine (GEL) in heated distilled water (20 min at 60 °C), respectively. The chitosan solution was homogenized using a homogeniser (IKA Ultra turax T18, Staufen, Germany) for complete biopolymer dissolving. The chitosan-only coating was used directly after preparation. In addition, the chitosan-gelatine (CS/GEL) formulation was obtained by mixing the individual solutions in a ratio of 25:75, followed by stirring for 20 min to ensure homogeneity. Subsequently, either 0.5% (*m*/*v*) sea fennel essential oil or 20% (*v*/*v*) rosemary extract was added. Following the addition of the active compounds, the dispersions were further mixed on a magnetic stirrer. Samples containing essential oil were additionally homogenized using the homogeniser to improve dispersion quality. The blank control consisted of an aqueous solution containing the same concentration of acetic acid as that used for the preparation of the coating formulations but without biopolymers. No gelatin-only treatment was included, as the objective of the study was to assess the effectiveness of the final CS/GEL coating formulations intended for food preservation applications. The abbreviations and formulations which were prepared and then tested on fish samples are given in Table 1, while the whole process is presented in Figure S1.

#### 2.2.3. Preparation of Fish Samples

Fresh sardines (*Sardina pilchardus*) and gilthead seabream fillets (*Sparus aurata*) were transported on ice from the provider to the laboratory to keep them fresh. On the day of the catch, fishes were treated with biopolymer formulations. The fishes were immersed in the prepared dispersions (250 mL of coating solution, corresponding to either seven whole sardines or two gilthead seabream fillets per coating batch) for 2 min in plastic boxes, drained for 2 min and transferred to clean plastic crates for storage at 4 °C. Coating uptake was not determined in the present study due to experimental and measurement limitations. A total of 280 sardines (7 fishes × 8 treatments × 5 sampling days) and 80 gilthead seabream fillets (2 fillets × 8 treatments × 5 sampling days) were included in the experiment. At each sampling point, all fish belonging to the same treatment replicate (7 sardines or 2 gilthead seabream) were pooled and homogenized using a hand blender, and the resulting homogenates were used for further analysis. At each sampling day, sensory evaluation of sardine freshness was performed prior to further processing. The sardines were then washed, gutted, descaled and filleted. Sampling times were established according to prior experience and preliminary laboratory investigations.

The fatty acid composition of the fish used in this study was determined by gas chromatography of fatty acid methyl esters; the analytical procedure and results are given in the Supplementary Materials (Table S1).

#### 2.2.4. Sensory Analysis

Sensory evaluation of fish freshness was performed according to the Quality Index Method (QIM) for sardines as described by Garcia and Careche [30]. The method is based on the assessment of freshness-related sensory attributes. The evaluated attributes included general appearance (surface brightness and rigour status), eyes (cornea clarity, pupil appearance, and eye shape), gills (bloodiness of the gill cover, internal gill colour, and odour), abdomen integrity, ease of fillet separation from the vertebral column, and muscle appearance and firmness. On each sampling day, QIM evaluation was performed on 7 specimens from each experimental group by five trained assessors. The resulting score for each sampling day was calculated as the arithmetic mean of demerit points assigned by the evaluator for the samples inspected, and the end of shelf life was determined when the maximum score of 29 demerit points was reached. Since QIM is not suitable for freshness assessment of fish fillets, sensory analysis was not conducted on gilthead seabream samples. Instead, the quality evaluation of gilthead seabream was based on physicochemical and microbiological indicators, which are more appropriate for processed fish products.

#### 2.2.5. Lipid Oxidation

The degree of lipid oxidation was measured using the 2-thiobarbituric acid reactive substances (TBARS) test described by Bruna et al. [31] with some modifications. The TBA value is widely used as an index of lipid oxidation because it quantifies malondialdehyde content that are produced from the breakdown of hydroperoxides, which are the primary reaction products generated when polyunsaturated fatty acids react with oxygen [32].

Briefly, 5 g of fish sample was homogenized in 20 mL trichloroacetic acid (5%), and 10 mg of butylhydroxytoluene was added to avoid further oxidation. To ensure better homogenization, the mixture was homogenized using a high-speed homogenizer (IKA Ultra turax T18, Staufen, Germany) for 60 s. Afterwards, the samples were centrifuged at 1200 rpm and 4 °C for 10 min and filtered through Watman filter paper (nº 54). An aliquot of 4 mL was mixed with 4 mL of 0.02 M 2-thiobarbituric acid (TBA), and the tubes were placed in a heating block (Stuart SBH130D, Cole-Parmer Ltd., Stone, UK) and incubated at 100 °C for 1 h. After cooling, the absorbance was measured at 532 nm. The measurements were performed in duplicate and the results are expressed as mg malonaldehyde (MDA)/kg sample ± standard deviation. The calibration curve was prepared using the standard 1,1,3, 3-tetrametoxypropane (TMP) in different concentrations (12.5, 6.25, 3.13, 1.56 and 0.75 µM in 5% trichloroacetic acid).

#### 2.2.6. Proteolytic Index

The proteolytic index was analyzed according to a method described by Janči et al. [33]. The method is based on the quantification of the total number of amino acids present in the sample (expressed as L-leucine; Leu) after derivatization with Cd-ninhydrin. A 2 g fish sample was homogenized with 20 mL of ice-cold 0.01 M HCl using a homogenizer (IKA Ultra turax T18, Staufen, Germany) for 60 s on ice and centrifuged for 20 min at 10,000 rpm at 4 °C in a Rotina 380 R centrifuge (Hettich LabTechnology, Tuttlingen, Germany). A volume of 400 µL of the supernatant was taken and mixed with 800 µL of absolute ethanol and stored at room temperature for 30 min to precipitate the proteins. Then, the solutions were centrifuged at 12,000 rpm for 5 min in a MicroCL 21 microcentrifuge (ThermoFisher Scientific, Waltham, MA, USA). A total of 400 µL of the supernatant was withdrawn and mixed with 800 µL of Cd-ninhydrin reagent, heated in a heating block (Stuart SBH130D, Cole-Parmer Ltd., Stone, UK) for 5 min at 84 °C, cooled on ice (t = 15 min), and centrifuged at 12,000 rpm for 5 min. A calibration curve was established for each measurement day using standard Leu solutions. The proteolytic index was determined by measuring the absorbance at 490 nm using leucine (Leu) as a standard. The measurements were performed in duplicate and the results were expressed as mg of total amino acids per gram of sample.

#### 2.2.7. Determination of pH and Total Volatile Basic Nitrogen (TVB-N)

The muscle pH was determined according to the method described by Vyncke [34]. Briefly, 10 g of homogenized fish sample was suspended in 100 mL of distilled water and mixed. The pH was determined using a pH metre with glass electrode (Metrohm, Filderstadt, Germany).

The total volatile basic nitrogen content of the fish samples was determined by direct distillation as described by Antonacopoulos and Vyncke [35]. A 10 g sample was weighed and transferred to a distillation tube and 50 mL of distilled water and 1 g of magnesium oxide were added. The tube content was mixed and placed in a distillation unit (Kjeltec 2100, Hilleroed, Denmark). The distillate was collected in the receiver containing 25 mL of boric acid (4% *v*/*v*), and the distillation time was exactly 4 min. Afterwards, the distillate was titrated with 0.1 M hydrochloric acid until the colour changed (from green to purple). The measurements were performed in duplicate.

#### 2.2.8. Assessment of Microbial Activity

Microbiological analyses of fish samples, performed every second day in accordance with the applicable regulation, included the determination of total aerobic mesophilic bacteria, Enterobacteriaceae, *Salmonella* sp., and sulphite-reducing bacteria using standardized ISO methods.

Total aerobic mesophilic bacteria were enumerated on Plate Count Agar (Biolife, Milan, Italy) (PCA, HRN EN ISO 4833:2008) [36] after incubation at 30 °C for 24 h. Enterobacteriaceae were determined on Violet Red Bile Glucose (VRBG) agar (Biolife) (HRN EN ISO 21528-2:2017) [37] following incubation at 37 °C for 24 h. *Salmonella* spp. detection included selective enrichment in RVS broth (Biolife) at 41.5 °C for 24 h and isolation on Xylose Lysine Deoxycholate (XLD) agar (Biolife) (HRN EN ISO 6579-1:2017) [38] incubated at 37 °C for 24 h. *Listeria monocytogenes* was detected using selective enrichment in Fraser broth (Biolife) and isolation on Oxford agar (Biolife) (ISO 11290-1:2017) [39] incubated at 37 °C for 24–48 h. Sulphite-reducing bacteria were cultivated on sulphite agar (Biolife) (ISO 15213-1:2023) [40] after heat treatment, with incubation at 37 °C for 48 h.

Microbial counts were expressed as colony-forming units per gram of fish (CFU/g) ± standard deviation, while *Salmonella* sp. and *L. monocytogenes* results were reported qualitatively (presence/absence in 25 g). The measurements were performed in triplicate.

#### 2.2.9. Statistical Analysis

Statistical analyses were performed using one-way analysis of variance (ANOVA) and two-way ANOVA followed by Tukey’s multiple comparison test to determine significant differences among groups. All statistical analyses were carried out using XLSTAT-Pro software (version 7.5.3, Addinsoft, New York, NY, USA). Results are presented as mean values ± standard deviation, and differences were considered statistically significant at a confidence level of 95% (*p* < 0.05).

## 3. Results and Discussion

### 3.1. Sensory Analysis

**Figure 1:**
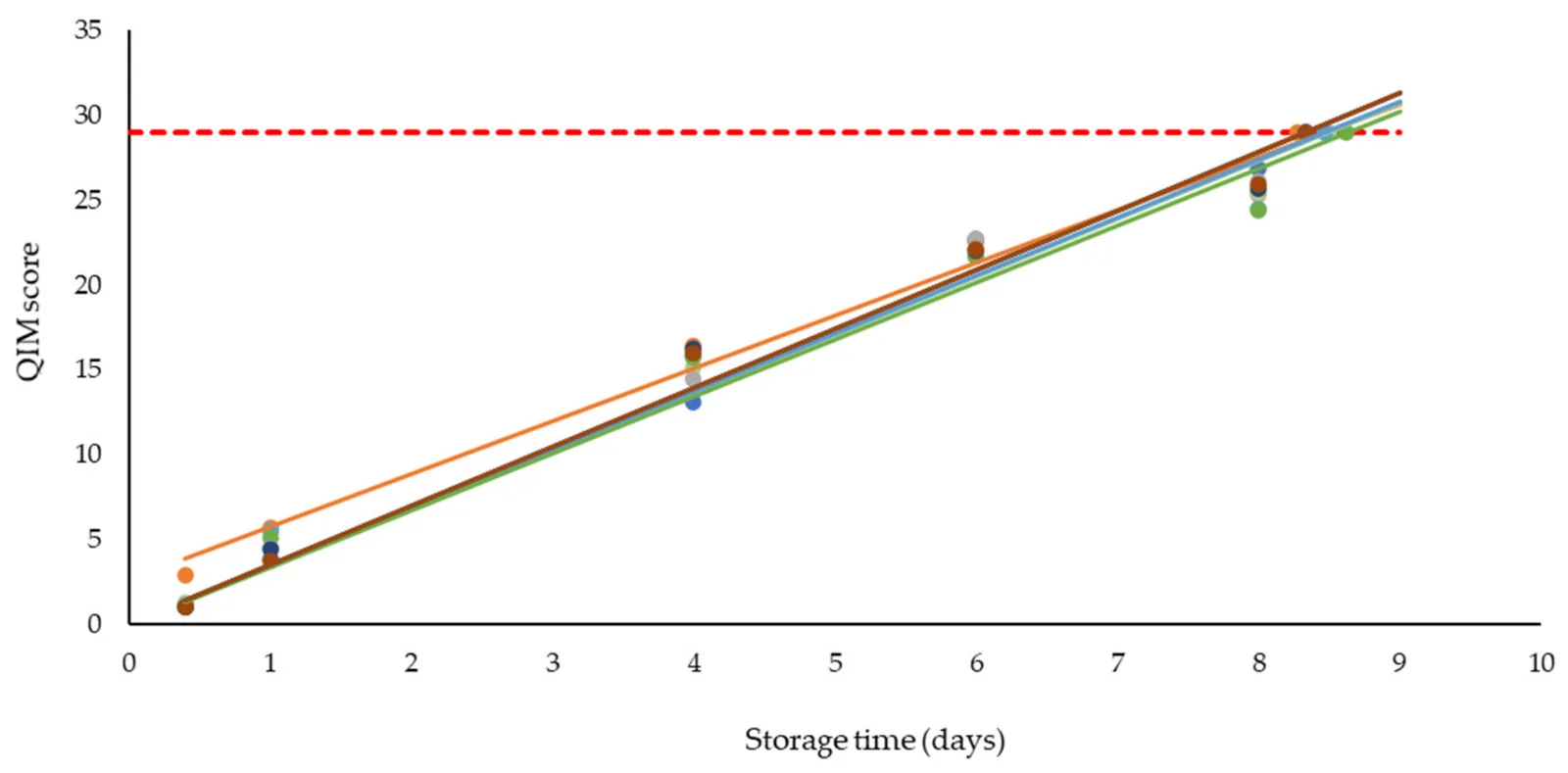
Linear QIM models for sardines (• control; • blank; • CS; • CS + SF; • CS + R; • CS/GEL; • CS/GEL + SF; • CS/GEL + R). (The red dotted line represents the end of shelf life which was de-fined as the point when the maximum QIM score was reached, corresponding to 29 demerit points).

Sensory evaluation of freshness was conducted to assess freshness loss in sardines and to determine the potential influence of edible coatings on shelf life. As mentioned in the Materials and Methods Section, the QIM model of sensory analysis is not suitable for application to gilthead seabream and for this reason the results are absent. All samples of sardines, regardless of treatment, reached the rejection limit of 29 demerit points on day 8 of storage, indicating the end of shelf life (Figure 1, Table 2). This suggests that applied coatings, including those enriched with bioactive compounds, did not have a significant impact on the extension of sardine shelf life. The linear QIM models (Table 2) exhibited an excellent fit, with high coefficients of determination (R^2^ = 0.9846–0.9964), confirming the reliability of the method for monitoring freshness. The slopes (a-values), which represent the rate of quality loss, were relatively similar across treatments, indicating that the overall progression of spoilage was only slightly affected by the coatings. Slightly lower slope values were observed for chitosan-based coatings, particularly for CS + R (a = 3.3627), suggesting a slower deterioration rate compared to the control (a = 3.4784). Although minor numerical differences were observed, particularly for chitosan-based coatings with rosemary extract, edible coatings did not result in a practically relevant extension of sardine shelf-life. These findings highlight the challenge of preserving small pelagic fish with fast spoilage kinetics and suggest that, while bioactive coatings may influence biochemical or microbiological parameters, their impact on sensory shelf life may remain limited. Aromatic plant extracts contain numerous volatile and phenolic compounds that may adversely affect the sensory attributes of coated foods, particularly odour and flavour. The selection of rosemary extract and sea fennel essential oil was based on their well-documented antioxidant and antimicrobial properties. The chemical profile of the rosemary extract used in this study was previously characterized and reported by Babić et al. [21]. The rosemary extract used was characterized by a high content of rosmarinic acid and aromatic terpenes such as eucalyptol, camphor, and borneol, while sea fennel contains a complex volatile profile dominated by limonene, γ-terpinene, sabinene, and α-pinene. There is also available data on the composition of the essential oil that was characterized by a predominance of limonene (>60%), followed by sabinene (~16%) and lower levels of γ-terpinene, α-pinene, cis-β-ocimene, and linalool. These compounds contribute characteristic citrus, herbal, and resinous notes which, depending on their concentration and interaction with the fish matrix, may affect odour perception and consequently influence sensory shelf-life assessment. This outcome is consistent with the study objective, emphasizing the need to evaluate coating performance across different fish species and preservation mechanisms.

### 3.2. Lipid Oxidation

Lipid oxidation is a factor contributing to fish deterioration, and it was measured using the TBARS test. Thiobarbituric acid (TBA) measures the concentration of compounds responsible for off-flavours and off-odours, making it particularly important during the later stages of lipid oxidation. TBARS values indicate the degree of rancidity in a product, and the values above 3–4 mg MDA/kg are considered as an indication of quality deterioration [41].

**Figure 2:**
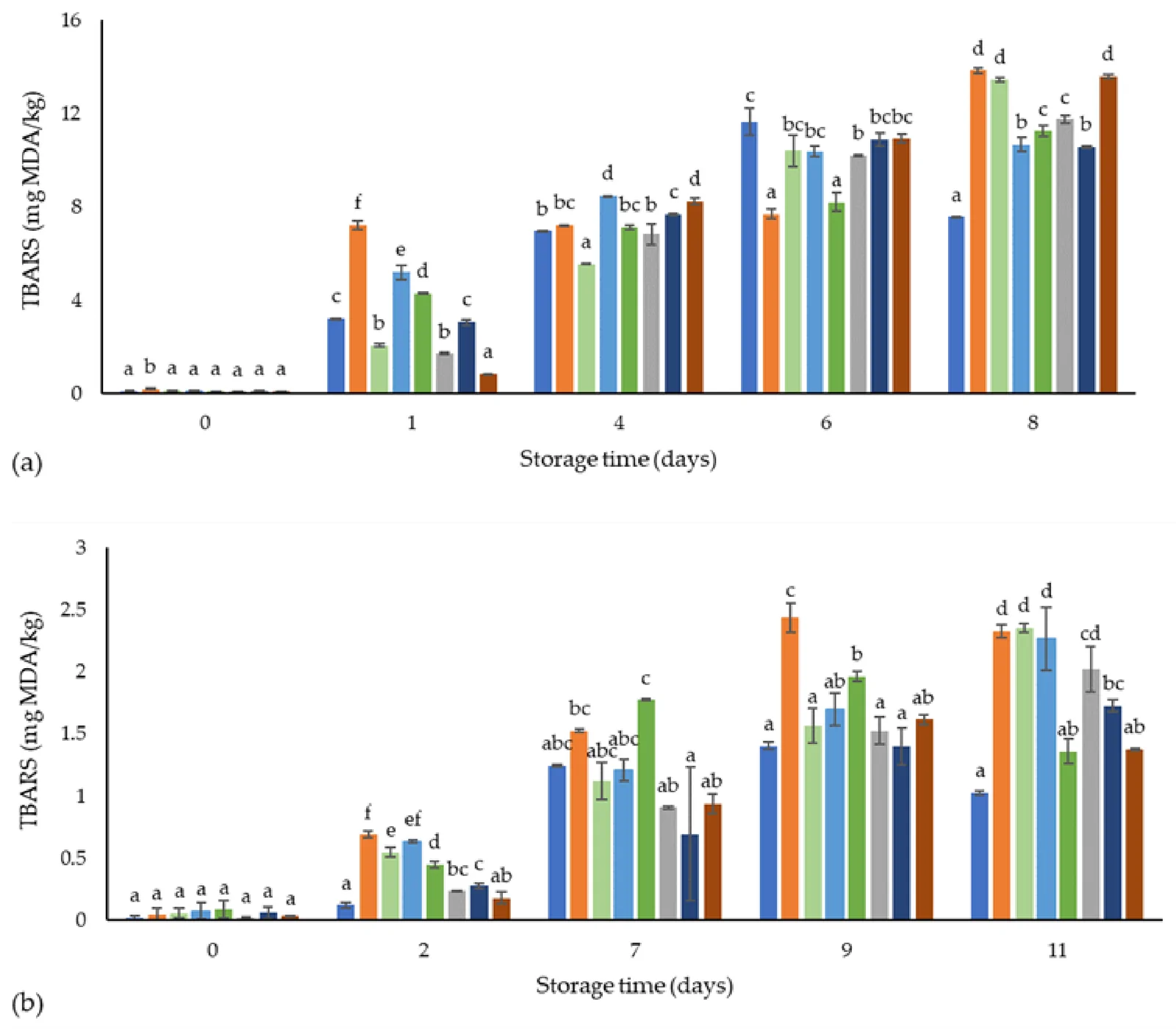
TBARS values obtained for (**a**) sardines and (**b**) gilthead seabream in different batches (■ control; ■ blank; ■ CS; ■ CS + SF; ■ CS + R; ■ CS/GEL; ■ CS/GEL + SF; ■ CS/GEL + R) (^a–f^ different lowercase superscript letters denote significant differences (*p* < 0.05) between values obtained for different treatments at the same sampling day).

Figure 2 shows the results of the TBARS test obtained for sardines and gilthead seabream for all treatments during the storage. The initial TBARS values ranged from 0.08 to 0.20 mg MDA/kg for sardines, and from 0.02 to 0.08 mg MDA/kg for gilthead seabream. Lipid oxidation, expressed as TBARS values, increased progressively during refrigerated storage in both sardines and gilthead seabream, confirming the expected deterioration of fish lipids over time. However, clear differences were observed between treatments and between fish species. In sardines, a rapid increase in TBARS was observed, reaching high values by day 8 (7.56–13.84 mg MDA/kg), reflecting the high lipid content and susceptibility of this species to oxidative spoilage. In contrast, gilthead seabream exhibited substantially lower TBARS values throughout storage (maximum 2.5 mg MDA/kg), confirming its greater oxidative stability. TBARS values were consistently higher in sardines than in gilthead seabream, which can be explained by differences in both the amount and the composition of their lipids. The average lipid content is approximately 6.42% in sardine [42] and 1.88% in gilthead seabream [43]. In addition, the fatty acid profiles of the fish used in this study (Table S1) showed that PUFA accounted for 43.6% of total fatty acids in sardine, compared with 30.2% in gilthead seabream. Sardine lipids were dominated by the highly unsaturated n-3 fatty acids DHA (26.6%) and EPA (11.7%), which together accounted for 38.3% of total fatty acids. In gilthead seabream, EPA and DHA accounted for 14.4%, while monounsaturated fatty acids, mainly oleic acid (30.2%), were the dominant fraction (45.9%). The more highly oxidizable substrate explains the faster and more intensive formation of secondary oxidation products in sardine, and may also explain why the coatings were not able to limit lipid oxidation in this species. Unexpectedly, at the end of storage, the control batches (untreated fish) showed some of the lowest TBARS values, which is contrary to expectations. This apparent inconsistency can be explained by the well-documented non-linear behaviour of TBARS during storage. TBARS values may increase to a maximum and subsequently decrease as storage progresses due to the complex chemistry of secondary lipid oxidation products. This behaviour is related to the complex chemistry of secondary lipid oxidation products, particularly malondialdehyde (MDA), which is not a terminal oxidation product. After its formation, MDA can further participate in secondary reactions or be lost from the sample due to its volatility [44,45]. Accordingly, differences among treatments in the present study were primarily observed in the temporal evolution of TBARS values, including the timing and magnitude of peak oxidation, rather than in a consistent reduction in final TBARS levels. This is further confirmed by two-way ANOVA tests that were used to evaluate the effects of coating treatment, storage time, and their interaction on quality parameters of tested fish during refrigerated storage (Table 3 and Table 4). These results indicate that, under the applied experimental conditions, the edible coatings did not exert a clear inhibitory effect on lipid oxidation as assessed by TBARS, and that TBARS data should be interpreted with caution and in conjunction with other spoilage indicators. Similar TBARS values for sardines were reported by Janči et al. [33] who investigated the effect of different handling practices on fish quality.

Furthermore, regarding the effects of coatings, chitosan coatings showed some antioxidant potential, particularly when combined with bioactive compounds. Chitosan coatings without added bioactives sometimes exhibited higher TBARS values compared to enriched formulations, and blending chitosan with gelatin (CS/GEL) did not systematically enhance oxidative stability, indicating that polymer combination did not produce a clear synergistic effect in terms of lipid oxidation control. However, the results were not consistent. In sardines, the lowest TBARS values were observed for the CS/GEL + sea fennel essential oil treatment, while in gilthead seabream the most effective treatment was CS/GEL + rosemary extract. In general, coatings containing rosemary extract tended to show stronger and more consistent antioxidant effects, likely due to the higher solubility of the aqueous extract of rosemary compared to that of the essential oil whose solubility is probably much reduced. Sea fennel essential oil exhibited a moderate but less consistent protective effect. These findings were different from those of previous studies, where significant reductions in TBARS values were reported for similar systems. For example, Kanelaki et al. [46] observed a strong antioxidant effect of gelatin film with the addition of 2% (*v*/*v*) of commercially produced rosemary extract on sardine fillets during short-term storage of 4 days. Similarly, reduced TBARS values were obtained for gelatin with the addition of 1, 1.5 and 2% (*v*/*v*) of cinnamon oil applied on rainbow trout [47], chitosan–gelatin with 1% (*m*/*v*) of clove oil on tuna fillets [48], and for the incorporation of 1% (*m*/*Vv*) thyme essential oil into chitosan coating applied on rainbow trout [49]. For gilthead seabream, Izci et al. [50] showed TBARS values ranging from 0.35 to 1.50 mg MDA/kg during 20 days of storage, which is in accordance with the values obtained in this research. The authors concluded that a natural biopolymer such as chitosan has the potential to extend the shelf life of seabream [50]. Similarly, studies on tambaqui fillets [7], salmon [32], and mullet fish steaks [51] showed that chitosan coatings reduced lipid oxidation and TBARS values during storage by 38.63, 18.18 and 82.26%, respectively. The discrepancies between these studies and the present results may be attributed to differences in fish species, lipid composition, storage duration, coating formulations, and especially the intensity of oxidative processes. In highly perishable species such as sardines, the rapid progression of lipid oxidation may limit the effectiveness of edible coatings, particularly over extended storage periods.

### 3.3. Proteolytic Index

Proteolytic enzymes are naturally present in the muscle tissue and visceral organs of fish. Following the death of the fish, these endogenous enzymes remain active and initiate protein breakdown. This enzymatic activity represents a major mechanism of post-mortem biochemical deterioration during storage and processing [52,53].

However, proteolysis contributes significantly to the deterioration of sensory and physicochemical quality in fish products. The degradation of structural muscle proteins results in textural softening, loss of firmness, and the formation of undesirable flavour compounds [54]. In addition, inadequate storage conditions accelerate enzymatic protein degradation in whole fish. Progressive hydrolysis of muscle proteins increases their solubility and disrupts the integrity of the myofibrillar network, leading to weakening of the muscle structure and increased water release [52].

**Figure 3:**
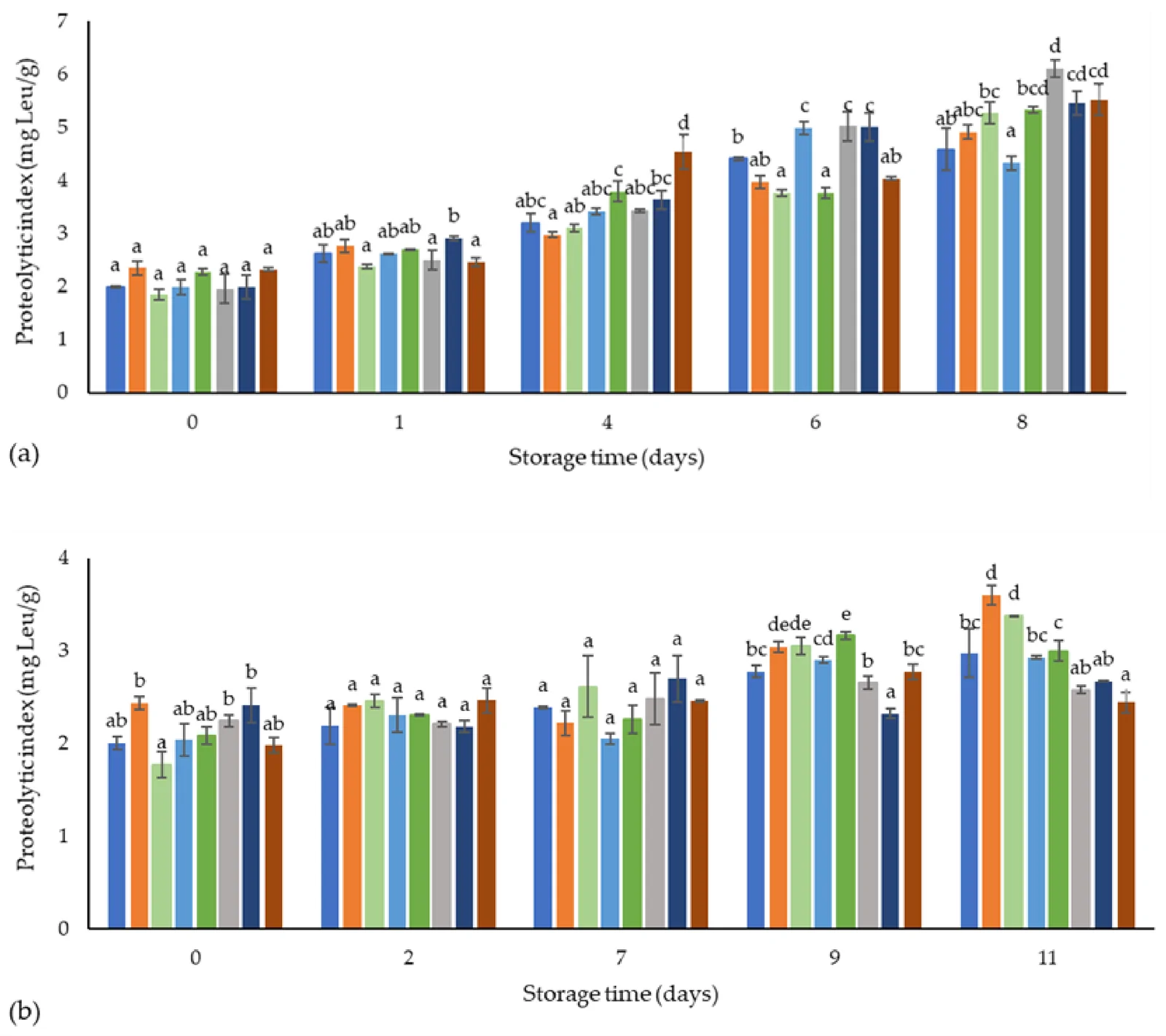
Results of proteolytic index during storage of: (**a**) sardines and (**b**) gilthead seabream in different batches (■ control; ■ blank; ■ CS; ■ CS + SF; ■ CS + R; ■ CS/GEL; ■ CS/GEL + SF; ■ CS/GEL + R) (^a–e^ different lowercase superscript letters denote significant differences (*p* < 0.05) between values obtained for different treatments at the same sampling day).

The results of the proteolytic index determined during the storage are presented in Figure 3. No significant difference between the treatments occurred at the day of fishing for sardines (1.84–2.34 mg Leu/g), while in gilthead seabream (1.77–2.43 mg Leu/g) there were indicative changes. During the storage, an increase in proteolytic index was observed, due to the progress in proteolysis (Table 3 and Table 4). Also, it can be noticed that the final values of the proteolytic index at the end of the storage for sardines were higher (4.32–6.10 mg Leu/g) than the values obtained for gilthead seabream (2.57–3.60 mg Leu/g). Janči et al. [33] reported similar values for sardines. The authors found that PI ranged from 1.78 to 1.87 mg Leu/g at the beginning of the study, reaching up to 2.50 mg Leu/g after 7 days of storage at 0 °C. Reported values were slightly lower than the ones obtained in the present research (3.40 mg Leu/g).

### 3.4. Determination of pH and Total Volatile Basic Nitrogen (TVB-N)

**Figure 4:**
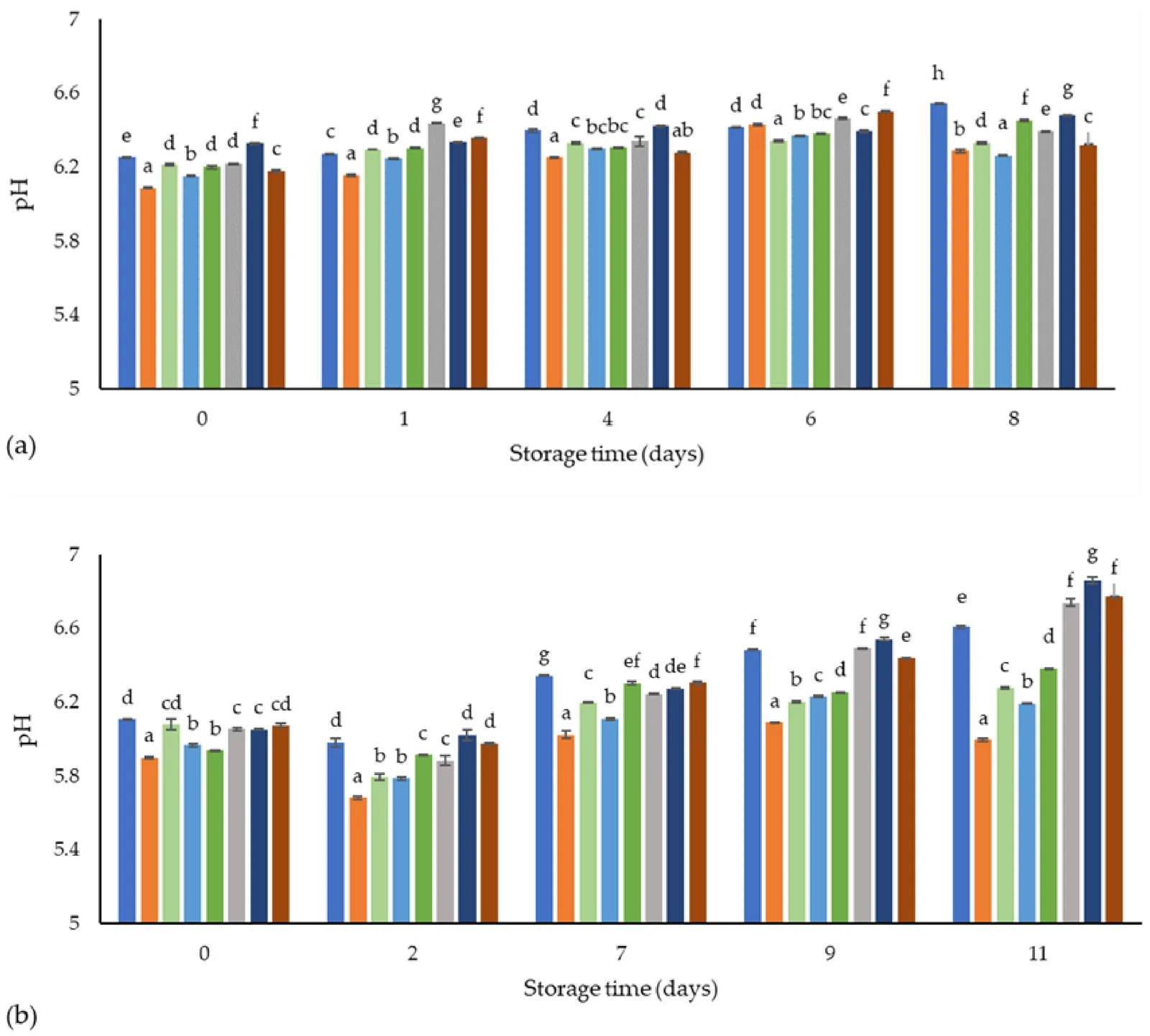
Changes in pH value during storage of: (**a**) sardines and (**b**) gilthead seabream in different batches (■ control; ■ blank; ■ CS; ■ CS + SF; ■ CS + R; ■ CS/GEL; ■ CS/GEL + SF; ■ CS/GEL + R) (^a–g^ different lowercase superscript letters denote significant differences (*p* < 0.05) between values obtained for different treatments at the same sampling day).

According to Aref et al. [51], pH is also an important indicator of freshness of fish products. Also, the pH post-mortem value can vary and depend on season, species, and other factors [55]. The changes in pH values of the treated fish samples are shown in Figure 4, while interactions with time are shown in Table 3 and Table 4. The initial pH values were from 6.25 to 6.54 for sardines, and from 5.98 to 6.48 for gilthead seabream which is in accordance with data from the literature [56,57]. A slight increase was observed in all treatments and for both fish samples with significant differences between the treatments. At the end of the storage, the pH values of the control were generally higher for both fish types, compared to the coated samples, except in gilthead seabream on day 11, where CS/GEL, CS/GEL + SF and CS/GEL + R exhibited pH values higher than the control. Similar findings were observed in the literature for grey mullet [51] and sardines [58], while higher pH values were reported for salmon [32] and filleted tilapia [55]. The slower increase in pH observed in coated samples (although not in all samples, as mentioned earlier) as storage progressed may be associated with reduced microbial and proteolytic activity. Chitosan alone was shown to have a stronger effect than when blended with gelatine, and differences among treatments were more pronounced in gilthead seabream when parameters were interpreted alone. However, two-way ANOVA showed that treatment x time was significant for both fish species. As spoilage microorganisms metabolize nitrogenous compounds, alkaline metabolites such as ammonia and amines are generated, leading to an increase in pH. By limiting microbial growth, chitosan coatings may have slowed the production of these compounds and contributed to maintaining more stable pH values during storage.

Total volatile basic-nitrogen (TVB-N) is a widely used parameter to evaluate the fish freshness and spoilage because it is closely associated with microbial activity and protein degradation during storage [50,51]. This parameter quantifies volatile nitrogenous compounds, including ammonia and primary, secondary and tertiary amines, which are mainly generated through the enzymatic degradation of proteins and non-protein nitrogenous compounds by endogenous enzymes and spoilage microorganisms. The accumulation of these compounds reflects the progression of spoilage and can therefore be used as an indicator of fish quality deterioration [32].

**Figure 5:**
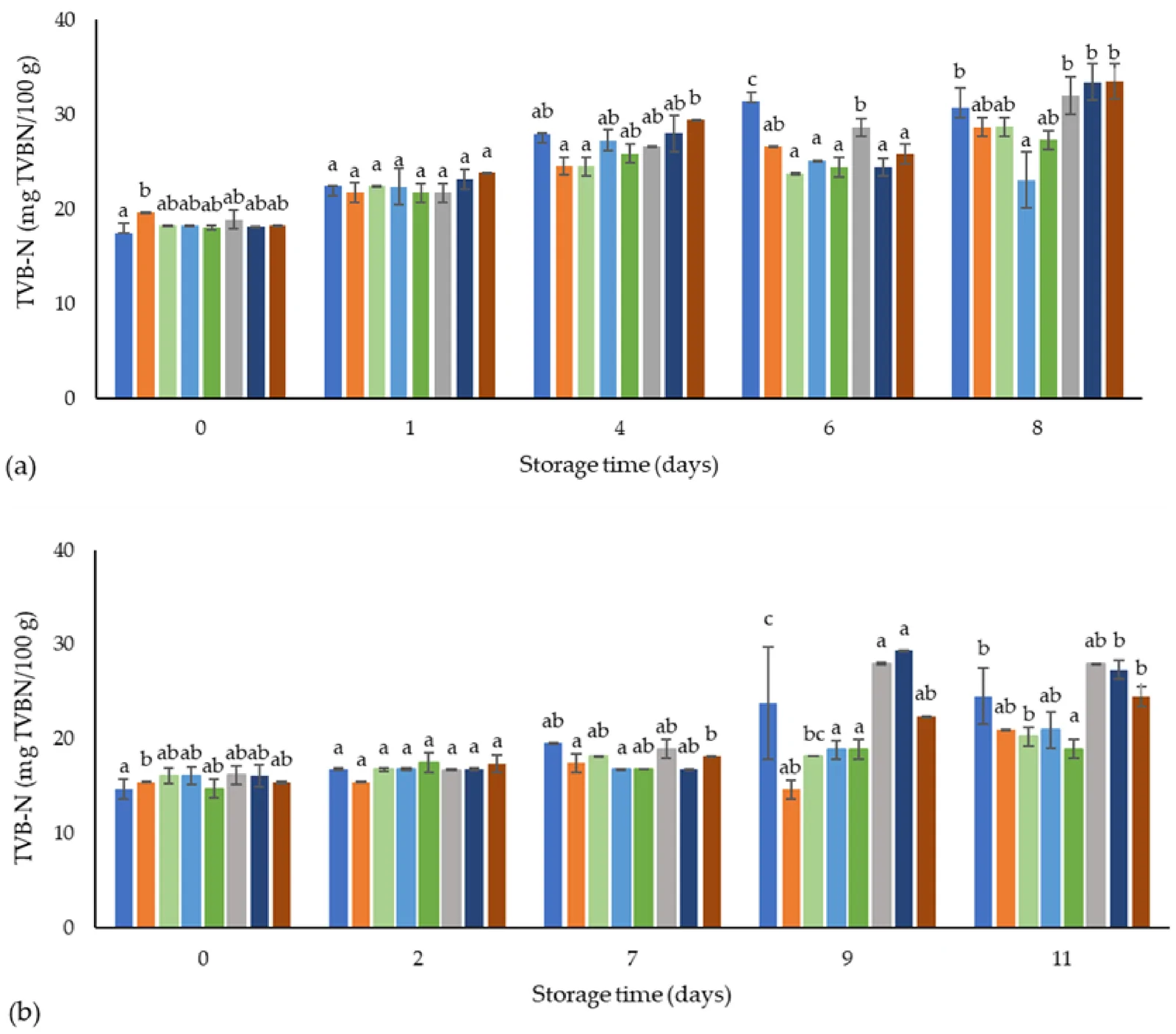
TVB-N content during storage of: (**a**) sardines and (**b**) gilthead seabream in different batches (■ control; ■ blank; ■ CS; ■ CS + SF; ■ CS + R; ■ CS/GEL; ■ CS/GEL + SF; ■ CS/GEL + R) (^a–c^ different lowercase superscript letters denote significant differences (*p* < 0.05) between values obtained for different treatments at the same sampling day).

The TVB-N results are presented in Figure 5. ranged from 17.50 to 19.61 mg/100 g for sardine samples and from 14.74 to 16.12 mg/100 g for gilthead seabream samples, confirming the good initial quality and freshness of the fish. During refrigerated storage, TVB-N values increased, reaching final values of 23.04–33.43 mg/100 g for sardines and 18.93–24.54 mg/100 g for gilthead seabream. The more pronounced increase observed in sardines may be associated with their higher lipid content and greater susceptibility to biochemical and microbial spoilage processes.

Furthermore, it can be noticed that the chitosan edible coatings with sea fennel oil or rosemary extract resulted in lower TVB-N values than the chitosan–gelatin coatings during the storage of both fish samples. Since volatile basic nitrogen compounds are largely produced through microbial and enzymatic degradation of nitrogen-containing constituents, inhibition of microbial growth can slow the formation of ammonia, trimethylamine, and other volatile spoilage metabolites. Consequently, chitosan-coated fish exhibited a delayed accumulation of TVB-N compared with the controls. The results for chitosan samples with sea fennel and rosemary, compared to CS/GEL as supporting matrix, also suggest that the chitosan matrix may provide a more effective carrier for bioactive compounds, potentially enhancing their antimicrobial and antioxidant action in the fish system. Similar observations were reported by Santos et al. [59], who found superior preservation effects of chitosan-based coatings in pirarucu fillets. The influence of polymer structure plays an important role [60,61,62,63] and is further explained later in the manuscript. In contrast to the obtained results, the available literature reported a more pronounced reduction in TVB-N values following the application of edible coatings and bioactive compounds. For example, Sathish Kumar et al. [48] investigated a chitosan and gelatin edible coating, either alone or combined with clove essential oil for the preservation of tuna fillets. After 12 days of storage, tuna fillets treated with an active chitosan–gelatin film had substantially lower TVB-N values compared with the untreated control (the reported values are 15.00 mg/100 g for the application of the active coating and 54.00 mg/100 g for the control). Likewise, Nowzari et al. [18] reported significant inhibition of TVB-N formation in rainbow trout coated with chitosan–gelatin films. Nevertheless, the TVB-N values obtained in the present study are comparable to those reported by Izci et al. [50], who evaluated the combined effect of chitosan coating and vacuum packaging on gilthead seabream. In that study, TVB-N values ranged from 14 to 27 mg/100 g throughout 20 days of refrigerated storage, which is similar to the range observed here. These similarities may be related to species-specific spoilage characteristics and differences in storage conditions, coating composition, and the effectiveness of the incorporated bioactive compounds. Overall, these findings highlight that the effectiveness of edible coatings in controlling TVB-N accumulation depends not only on the coating properties but also on the intrinsic characteristics of the fish species and the dominant spoilage pathways.

From Table 3 it can be concluded that storage time significantly affected all measured parameters during sardine storage (*p* < 0.0001). Significant effects of coating treatment on sardines were observed for PI, pH, and TVB-N (*p* < 0.01), whereas no significant treatment effect was detected for TBARS (*p* = 0.677). Significant treatment × storage time interactions were observed for PI (*p* = 0.001), pH (*p* < 0.0001), and TVB-N (*p* < 0.0001), indicating that the effect of the coatings varied throughout storage. In contrast, the interaction was not significant for TBARS (*p* = 0.055).

Two-way ANOVA (Table 4) revealed that in gilthead seabream, sample type and storage time significantly affected all evaluated quality parameters (TBARS, PI, TVB-N, L*, a*, and b*) (*p* < 0.001). Similar to sardines, storage time was generally the dominant factor, as indicated by the highest F-values for most parameters. A Treatment × Time interaction was observed for PI, TVB-N, L* and a* (*p* < 0.0001), indicating that the effect of storage time depended on the treatment applied and that coatings influenced the rate or extent of quality changes over time. In contrast, the Treatment × Time interaction was not significant for TBARS (*p* = 0.101) and b* (*p* = 0.309)**, indicating that although treatments and storage time individually influenced lipid oxidation and yellowness, the temporal pattern of change was similar across all samples.

### 3.5. Microbiological Analysis

In the legal framework of the European Union (EC Regulation No. 2073/2005) [64], when it comes to fish and fish products, the primary focus is on specific pathogens (such as *Listeria monocytogenes* and *Salmonella*) and chemical spoilage indicators (histamine and TVB-N), while the total number of aerobic mesophilic microorganisms is generally used as a criterion for the hygiene of the production process. The results of the microbiological analysis are presented in Table 5 for sardines and in Table 6 for gilthead seabream coating.

Although the initial microbial load in the control and blank samples was around 10^4^ CFU/g, this did not result in faster spoilage, as the total microbial count includes bacteria not directly responsible for spoilage. These results coincide with a study written by Parlapani et al. [65], where the initial population of the total mesophilic microflora ranged between 4.58 and 4.70 log CFU/g. The initial aerobic bacteria on sardines (fresh, newly caught fish) represent the natural microflora of their skin, gills and digestive tract, and the number and the composition directly depend on the cleanliness of the sea, water temperature and the way they are handled after catching. Fish spoilage is primarily driven by specific spoilage organisms (SSOs), whose presence and dominance have a greater impact than the total microbial load [65].

The shelf life of the control sample can be considered spoiled on day eight, while the CS + SF treatment showed the best performance, with no detectable microorganisms even after eight days. This enhanced effect can be attributed to the combined action of chitosan and *Crithmum maritimum* essential oil. The chemical composition and antimicrobial properties of the sea fennel essential oil and rosemary extract incorporated into the coatings have been previously characterized and reported by Babić et al. [21], demonstrating their potential as natural antimicrobial agents. Chitosan exhibits antimicrobial activity by interacting with negatively charged bacterial cell membranes, causing cell damage and growth inhibition, while also forming a protective film on the fish surface that limits oxygen availability. The essential oil contributes additional antimicrobial and antioxidant effects, leading to a combined effect that is particularly relevant for fatty fish species such as sardines, where high lipid content accelerates oxidative deterioration during refrigerated storage [66]. Also, Pedreiro et al. [67] proved that sea fennel essential oil represents a valuable source of bioactive compounds with the potential of antioxidant activity. Analysis of the chemical composition showed that essential oil consists almost entirely of monoterpenes, with γ-terpinene (37.2%), sabinene (21.2%) and thymyl methyl oxide (16.4%) being the most abundant. The high content of these terpenes indicates significant antioxidant activity of sea fennel essential oil [67].

Other treatments showed similar levels of total aerobic mesophilic bacteria (approximately 10^4^ CFU/g) after eight days of storage; none of them exceeded the maximum limit of 10^5^ CFU/g established by ICMSF [68].

Compared to sardines, gilthead seabream showed a lower initial microbial load, with microorganisms detected in the control sample only on day seven, when the maximum acceptable level of 10^5^ CFU/g was reached.

The blank sample maintained its shelf life until day eleven, indicating that the initial rinsing step was effective in removing part of the microbial load and slowing spoilage. The highest antimicrobial efficiency was the chitosan solution (CS), confirming its previously described antimicrobial effect, with the total aerobic mesophilic bacterial count reaching 6.30 × 10^4^ ± 0.52 × 10^3^ CFU/g on day eleven of storage. In some samples, the presence of enterobacteria was observed, with counts increasing over time (Table 6). Since these are not part of the typical natural microflora of fish, their presence likely indicates contamination during handling or sampling procedures.

*Salmonella* sp., *Listeria monocytogenes*, and sulphite-reducing clostridia were not detected in any of the sardine or gilthead seabream samples.

Since the physicochemical properties of the coatings were not determined in this study, the following explanations are based on the literature and on the trends observed in the present results, and should be regarded as hypotheses. First, the better performance of the chitosan coating compared with the chitosan–gelatin blend may be related to the composition of the blend. It is possible that some of the protonated amino groups of chitosan that are responsible for its antimicrobial activity were bonded to gelatin, so this would reduce the number of free cationic sites available for antimicrobial action [60,61,62]. In addition, since gelatin is a protein that can be hydrolysed by proteolytic spoilage bacteria and may therefore serve as an additional nutrient source at the fish surface, this could explain why, in gilthead seabream fillets, aerobic mesophilic counts in CS/GEL-coated samples on day 11 were similar to or higher than those of the control, and why Enterobacteriaceae were detected only in the control and in CS/GEL and CS/GEL + SF samples (Table 6).

Regarding the interaction between coating and fish, the coatings were in direct contact with the muscle in gilthead seabream fillets, whereas in whole sardines they were in contact with the skin. In whole, ungutted sardines, the sensory changes assessed by QIM, such as changes in the eyes, gills and abdomen integrity, are largely driven by autolytic processes, including the activity of digestive enzymes [63], which a surface coating cannot effectively control. This may explain why the complete inhibition of microbial growth by the CS + SF coating did not extend the sensory shelf life of sardines.

### 3.6. Limitations of the Study

Some limitations should be considered when interpreting the results. First, the physicochemical properties of the coatings (viscosity, thickness, microstructure, water vapour and oxygen barrier properties) and the coating uptake were not determined, as the study focused on the quality of the coated fish. The viscosity of the coating solution affects the amount and thickness of the coating retained on the fish surface, while the microstructure of the polymer network affects the diffusion of oxygen and water and the release of active compounds. Therefore, the mechanisms discussed should be confirmed by direct measurements. These are planned in future work and will include rheological measurements of the coating solutions, analysis of coating thickness and structure, determination of barrier properties, and release of active compounds into food simulants. Second, since the composition of sea fennel essential oil varies considerably with chemotype and origin [21,67], the observed effects should be attributed to the specific formulations used rather than generalized to all sea fennel essential oils or rosemary extracts. Third, the two fish groups differed not only in species but also in sample form (whole sardines vs. seabream fillets) and origin (wild vs. farmed), which is also reflected in their fatty acid profiles (Table S1). The differences between species should therefore be interpreted as trends reflecting the combined effect of biological characteristics, sample form and origin.

## 4. Conclusions

The effectiveness of chitosan and chitosan–gelatin edible coatings was species- and biopolymer-dependent, highlighting the importance of spoilage mechanisms and intrinsic composition of the tested fish as well as the importance of perfect matching combinations for application.

In sardines, none of the coatings significantly extended sensory shelf life, as all samples reached the QIM rejection limit on day 8, and none significantly limited lipid oxidation (TBARS up to 13.84 mg MDA/kg). Although the chitosan coating with sea fennel essential oil kept aerobic mesophilic bacteria below the detection limit throughout storage, this did not extend the shelf life of this lipid-rich pelagic fish.

Chitosan alone kept aerobic mesophilic counts below 10^5^ CFU/g until day 11, whereas the control exceeded this limit by day 7. In gilthead seabream, which had a lower proportion of EPA and DHA than sardines, lipid oxidation remained low (TBARS ≤ 2.5 mg MDA/kg). Chitosan-based coatings were more effective. Those containing sea fennel essential oil or rosemary extract performed better than chitosan–gelatin coatings, indicating that the monopolymer system is a more suitable carrier for the tested bioactive compounds.

Storage time was the main factor affecting quality changes in both sardines and gilthead seabream, significantly influencing all evaluated parameters (*p* < 0.001). Coating treatments significantly affected several spoilage indicators, including PI, pH, and TVB-N in sardines, while all measured parameters in seabream were significantly influenced by sample type. Significant Treatment × Time interactions for PI, TVB-N, pH (sardines) and PI, TVB-N, L*, and a* (seabream) indicate that coating effectiveness varied with storage time. In both species, coated samples generally showed lower pH and TVB-N values than the controls, and chitosan coatings performed better than chitosan–gelatin coatings in limiting proteolysis, TVB-N formation and microbial growth.

Also, these results indicate that microbial inhibition alone may not be sufficient to substantially extend the shelf life of sardines. While the chitosan and sea fennel essential oil coating successfully maintained microbial counts below detectable levels, sensory deterioration continued during storage, emphasizing the rapid spoilage kinetics and high perishability of small pelagic fish.

These results indicate that edible coatings for fish should be optimized for the target species, with particular emphasis on antioxidant protection in lipid-rich species. Also, due to differences in sample species, source and form, the observed differences should be interpreted as trends rather than exclusively species-specific responses, as variations in biological characteristics, muscle composition, and commercial origin may also have contributed to the distinct spoilage patterns and coating performance.

## Figures and Tables

**Table 1:** List of abbreviations and explanations of prepared coating solutions.

Abbreviation	Formulation
C	control, sardines or gilthead seabream without treatment
B	blank, sardines or gilthead seabream treated with 1% acetic acid
CS	chitosan dispersion
CS + SF	chitosan dispersion with sea fennel essential oil
CS + R	chitosan dispersion with rosemary extract
CS/GEL	chitosan/gelatin dispersion
CS/GEL + SF	chitosan/gelatin dispersion with sea fennel essential oil
CS/GEL + R	chitosan/gelatin dispersion with rosemary extract

**Table 2:** Slope (a), coefficient of determination (R^2^) and theoretical shelf life for linear QIM models for sardines.

Formulation	Equation	a	R^2^	Theoretical Shelf Life (Days)
C	y = 3.4784 x	3.4784	0.9964	8.33
B	y = 3.5056 x	3.5056	0.9846	8.27
CS	y = 3.4122 x	3.4122	0.9915	8.49
CS + SF	y = 3.4229 x	3.4229	0.9847	8.47
CS + R	y = 3.3627 x	3.3627	0.9874	8.62
CS/GEL	y = 3.4813 x	3.4813	0.9946	8.33
CS/GEL + SF	y = 3.4795 x	3.4795	0.9912	8.33
CS/GEL + R	y = 3.4854 x	3.4854	0.9932	8.32

**Table 3:** Results of two-way ANOVA evaluating the effects of coating treatment, storage time, and their interaction on quality parameters of sardines during refrigerated storage.

Parameter	Effect	df	F-Value	*p*-Value	Significance
TBARS	Treatment	7	0.693	0.677	NS
	Time	4	118.552	<0.0001	***
	Treatment × Time	28	1.724	0.055	NS
PI	Treatment	7	3.747	0.003	**
	Time	4	205.31	<0.0001	***
	Treatment × Time	28	2.912	0.001	**
pH	Treatment	7	58.481	<0.0001	***
	Time	4	214.973	<0.0001	***
	Treatment × Time	28	13.847	<0.0001	***
TVB-N	Treatment	7	11.364	<0.0001	***
	Time	4	234.534	<0.0001	***
	Treatment × Time	28	5.956	<0.0001	***

Residual error: df = 41 for all models. Significance codes: NS, not significant (*p* ≥ 0.05); ** *p* < 0.01; *** *p* < 0.001.

**Table 4:** Results of two-way ANOVA evaluating the effects of coating treatment, storage time, and their interaction on quality parameters of gilthead seabream during refrigerated storage.

Parameter	Effect	df	F-Value	*p*-Value	Significance
TBARS	Treatment	7	4.855	<0.001	***
	Time	4	97.874	<0.0001	***
	Treatment × Time	28	1.542	0.101	NS
PI	Treatment	7	8.552	<0.0001	***
	Time	4	120.17	<0.0001	***
	Treatment × Time	28	8.089	<0.0001	***
pH	Treatment	7	63.766	<0.0001	***
	Time	4	275.014	<0.0001	***
	Treatment × Time	28	7.220	<0.0001	***
TVB-N	Treatment	7	32.703	<0.0001	***
	Time	4	173.416	<0.0001	***
	Treatment × Time	28	11.559	<0.0001	***
L*	Treatment	7	217.375	<0.0001	***
	Time	4	58.409	<0.0001	***
	Treatment × Time	28	28.055	<0.0001	***
a*	Treatment	7	5.892	<0.0001	***
	Time	4	34.054	<0.0001	***
	Treatment × Time	28	2.986	<0.0001	***
b*	Treatment	7	5.396	<0.0001	***
	Time	4	108.954	<0.0001	***
	Treatment × Time	28	1.128	0.309	NS

Residual error: df = 41 for TBARS, PI and TVB-N; df = 201 for L*, a* and b*. Significance codes: NS = *p* ≥ 0.05; *** *p* < 0.001.

**Table 5:** Results of microbiological analysis during sardine storage (CFU/g).

Storage Day	Treatment	Aerobic Mesophilic Bacteria	Enterobacteriaceae	*Salmonella* sp.	*Listeria monocytogenes*	Sulphite-Reducing Bacteria
	C	2.34 × 10^4^ ±0.20 × 10^4^	BDL	BDL	BDL	BDL
	B	3.24 × 10^4^ ± 0.32 × 10^4^	BDL	BDL	BDL	BDL
0	CS CS + SF CS + R CS/GEL CS/GEL + SF CS/GEL + R	BDL BDL BDL BDL BDL 7.20 × 10^3^ ± 0.50 × 10^3^	BDL BDL BDL BDL BDL BDL	BDL BDL BDL BDL BDL BDL	BDL BDL BDL BDL BDL BDL	BDL BDL BDL BDL BDL BDL
	C	3.24 × 10^4^ ±0.50 × 10^4^	BDL	BDL	BDL	BDL
	B	4.50 × 10^4^ ± 0.45 × 10^4^	BDL	BDL	BDL	BDL
1	CS CS + SF CS + R CS/GEL CS/GEL + SF CS/GEL + R	BDL BDL BDL BDL BDL 5.40 × 10^3^ ± 0.50 × 10^3^	BDL BDL BDL BDL BDL BDL	BDL BDL BDL BDL BDL BDL	BDL BDL BDL BDL BDL BDL	BDL BDL BDL BDL BDL BDL
	C	8.10 × 10^4^ ± 0.50 × 10^3^	BDL	BDL	BDL	BDL
	B	2.52 × 10^4^ ± 0.25 × 10^3^	BDL	BDL	BDL	BDL
4	CS CS + SF CS + R CS/GEL CS/GEL + SF CS/GEL + R	BDL BDL 1.80 × 10^3^ ± 0.10 × 10^3^ 1.80 × 10^3^ ± 0.15 × 10^3^ 3.60 × 10^3^ ± 0.35 × 10^3^ 7.20 × 10^3^ ± 0.50 × 10^3^	BDL BDL BDL BDL BDL BDL	BDL BDL BDL BDL BDL BDL	BDL BDL BDL BDL BDL BDL	BDL BDL BDL BDL BDL BDL
	C	9.00 × 10^4^ ±0.10 × 10^3^	BDL	BDL	BDL	BDL
	B	3.50 × 10^4^ ± 0.35 × 10^3^	BDL	BDL	BDL	BDL
6	CS CS + SF CS + R CS/GEL CS/GEL + SF CS/GEL + R	BDL BDL 3.60 × 10^3^ ± 0.35 × 10^3^ 3.60 × 10^3^ ± 0.50 × 10^3^ 5.20 × 10^3^ ± 0.50 × 10^3^ 9.00 × 10^3^ ± 0.50 × 10^3^	BDL BDL BDL BDL BDL BDL	BDL BDL BDL BDL BDL BDL	BDL BDL BDL BDL BDL BDL	BDL BDL BDL BDL BDL BDL
	C	6.48 × 10^5^ ±0.55 × 10^3^	BDL	BDL	BDL	BDL
	B	5.40 × 10^4^ ±0.52 × 10^3^	BDL	BDL	BDL	BDL
8	CS CS + SF CS + R CS/GEL CS/GEL + SF CS/GEL + R	3.60 × 10^3^ ±0.35 × 10^3^ BDL 1.08 × 10^4^ ±0.10 × 10^3^ 1.08 × 10^4^ ±0.10 × 10^3^ 2.88 × 10^4^ ±0.25 × 10^3^ 5.40 × 10^4^ ±0.55 × 10^3^	BDL BDL BDL BDL BDL BDL	BDL BDL BDL BDL BDL BDL	BDL BDL BDL BDL BDL BDL	BDL BDL BDL BDL BDL BDL

BDL below the detection limit of the analytical method.

**Table 6:** Results of microbiological analysis during gilthead seabream storage (CFU/g).

Storage Day	Treatment	Aerobic Mesophilic Bacteria	Enterobacteriaceae	*Salmonella* sp.	*Listeria monocytogenes*	Sulphite-Reducing Bacteria
	C	BDL	BDL	BDL	BDL	BDL
	B	BDL	BDL	BDL	BDL	BDL
0	CS CS + SF CS + R CS/GEL CS/GEL + SF CS/GEL + R	BDL BDL BDL BDL 9.00 × 10^2^ ± 0.50 × 10^2^ BDL	BDL BDL BDL BDL BDL BDL	BDL BDL BDL BDL BDL BDL	BDL BDL BDL BDL BDL BDL	BDL BDL BDL BDL BDL BDL
	C	BDL	BDL	BDL	BDL	BDL
	B	BDL	BDL	BDL	BDL	BDL
2	CS CS + SF CS + R CS/GEL CS/GEL + SF CS/GEL + R	BDL BDL BDL BDL 9.00 × 10^2^ ± 0.55 × 10^2^ BDL	BDL BDL BDL BDL BDL BDL	BDL BDL BDL BDL BDL BDL	BDL BDL BDL BDL BDL BDL	BDL BDL BDL BDL BDL BDL
	C	3.60 × 10^5^ ± 0.50 × 10^5^	1.80 × 10^3^ ± 0.10 × 10^3^	BDL	BDL	BDL
	B	3.60 × 10^4^ ± 0.22 × 10^3^	BDL	BDL	BDL	BDL
7	CS CS + SF CS + R CS/GEL CS/GEL + SF CS/GEL + R	9.00 × 10^2^ ± 0.10 × 10^2^ BDL BDL 4.50 × 10^3^ ± 0.55 × 10^3^ 1.80 × 10^3^ ± 0.10 × 10^3^ 9.00 × 10^2^ ± 0.50 × 10^2^	BDL BDL BDL BDL BDL BDL	BDL BDL BDL BDL BDL BDL	BDL BDL BDL BDL BDL BDL	BDL BDL BDL BDL BDL BDL
	C	8.46 × 10^8^ ± 0.10 × 10^8^	3.65 × 10^4^ ±0.20 × 10^3^	BDL	BDL	BDL
	B	3.40 × 10^4^ ± 0.50 × 10^3^	BDL	BDL	BDL	BDL
9	CS CS + SF CS + R CS/GEL CS/GEL + SF CS/GEL + R	2.34 × 10^4^ ± 0.25 × 10^3^ 1.17 × 10^5^ ± 0.20 × 10^3^ 1.65 × 10^5^ ± 0.20 × 10^3^ 9.54 × 10^8^ ± 0.10 × 10^7^ 1.99 × 10^9^ ± 0.20 × 10^9^ 3.69 × 10^8^ ± 0.20 × 10^8^	BDL BDL BDL 9.00 × 10^3^ ± 0.15 × 10^3^ 9.90 × 10^3^ ± 0.10 × 10^3^ BDL	BDL BDL BDL BDL BDL BDL	BDL BDL BDL BDL BDL BDL	BDL BDL BDL BDL BDL BDL
	C	2.70 × 10^10^ ± 0.25 × 10^10^	2.70 × 10^10^ ± 0.25 × 10^10^	BDL	BDL	BDL
	B	4.50 × 10^5^ ± 0.42 × 10^4^	BDL	BDL	BDL	BDL
11	CS CS + SF CS + R CS/GEL CS/GEL + SF CS/GEL + R	6.30 × 10^4^ ± 0.52 × 10^3^ 1.71 × 10^5^ ± 0.15 × 10^4^ 1.20 × 10^6^ ± 0.15 × 10^5^ 3.60 × 10^10^ ± 0.55 × 10^10^ 4.50 × 10^10^ ± 0.25 × 10^10^ 4.50 × 10^10^ ± 0.52 × 10^10^	BDL BDL BDL 1.85 × 10^5^ ± 0.22 × 10^4^ 4.50 × 10^5^ ± 0.50 × 10^4^ BDL	BDL BDL BDL BDL BDL BDL	BDL BDL BDL BDL BDL BDL	BDL BDL BDL BDL BDL BDL

BDL below the detection limit of the analytical method.

## Data Availability

The original contributions presented in this study are included in the article/Supplementary Materials. Further inquiries can be directed to the corresponding author.

## Supplementary Materials

The following supporting information can be downloaded at: https://www.mdpi.com/article/10.3390/foods15203578/s1, Reference cited in Supplementary Materials [69].
