# Supplementary material for: Application of Chitosan-Based Coatings Incorporating Sea Fennel Essential Oil and Rosemary Extract on the Chemical and Microbiological Characteristics of Sardine (Sardina pilchardus) and Gilthead Seabream (Sparus aurata)
Source: Foods. 2026 Oct 9;15(20):3578. doi: 10.3390/foods15203578 (PMC13703637; doi:10.3390/foods15203578)
Supplement: Supplementary file 1 [file foods-15-03578-s001.zip › foods-4573108-supplementary.pdf]

Supplementary

Article

Application of Chitosan-Based Coatings Incorporating Sea Fennel Essential Oil and Rosemary Extract on the Chemical and Microbiological Characteristics of Sardine (*Sardina pilchardus*) and Gilthead Seabream (*Sparus aurata*)

Tibor Janči [1], Tea Sokač Cvetnić [1], Iva Čanak [1], Petra Babić [1], Emili Vidan [1], Nasreddine Benbettaieb [2,3], Frédéric Debeaufort [2,3] and Mia Kurek [1,*]

Figure S1.

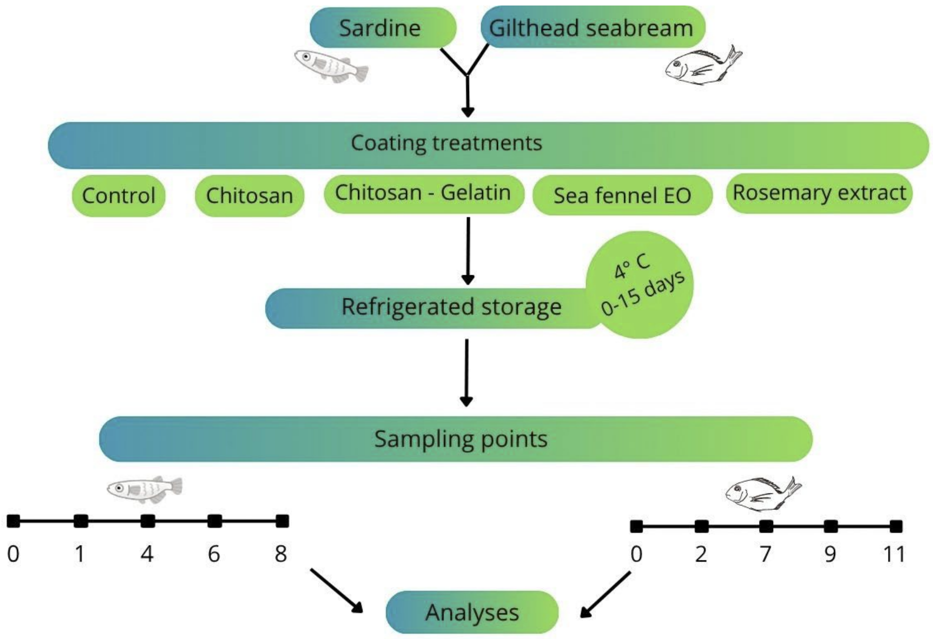

Analyses		Sardine	Gilthead seabream
Chemical	Lipid oxidation	+	+
	Proteolytic index	+	+
	Determination of pH	+	+
	Total Volatile Basic Nitrogen	+	+
Microbiological (Standardized ISO methods)	Total aerobic mesophilic bacteria	+	+
	Enterobacteriaceae	+	+
	Salmonella sp.	+	+
	Sulphite-reducing bacteria	+	+
Sensory	Quality Index Method (QIM)	+	-

List of abbreviations:

BDL – Below the detection limit of the analytical method
B – Blank; sardines or gilthead seabream treated with 1% acetic acid
CFU – Colony-forming units
C – Control; sardines or gilthead seabream without treatment
CS – Chitosan
CS/GEL – Chitosan-gelatin
GEL – Gelatin
MDA – Malondialdehyde
PI – Proteolytic index
QIM – Quality Index Method
R – Aqueous rosemary extract
SF – Sea fennel essential oil
TBARS – Thiobarbituric acid reactive substances
TVB-N – Total volatile basic nitrogen

Table S1. Fatty acid composition (% of total fatty acids) of sardine (*Sardina pilchardus*) and gilthead seabream (*Sparus aurata*) used in the study. Values are means ± standard deviation (n = 2).

**Fatty acid**	**% of total fatty acids**
	Sardines
**C12:0**	0.252±0.003
**C14:0**	8.006±0.062
**C14:1**	0.036±0.000
**C15:0**	0.8758±0.005
**C16:0**	20.659±0.093
**C16:1**	6.384±0.037
**C17:0**	0.997±0.002
**C17:1**	0.097±0.001
**C18:0**	5.006±0.016
**C18:1c**	10.717±0.298
**C18:2c**	1.996±0.009
**C18:3n6**	0.178±0.001
**C18:3n3**	1.222±0.012
**C20:0**	0.915±0.009
**C20:1**	1.218±0.008
**C20:2**	0.419±0.004
**C20:4n6**	1.259±0.013
**C20:3n3**	0.213±0.001
**C20:5n3**	11.691±0.028
**C22:1**	0.188±0.001
**C23:0**	0.113±0.002
**C24:0**	0.164±0.001
**C22:6n3**	26.593±0.009

**Fatty acid**	**% of total fatty acids**
	Gilthead seabream
**C14:0**	3.871±0.002
**c16:0**	16.369±0.018
**C16:1-9 cis**	6.215±0.006
**c18:0**	3.649±0.062
**C18:1-9 cis**	30.199±0.257
**C18:1-11 cis**	4.113±0.014
**C18:2-9, 12 cis**	11.845±0.043
**c18:3-9,12,15 cis**	2.804±0.009
**C18:4-6,9,12,15 cis**	1.199±0.005
**C20:1-11 cis**	3.146±0.020
**C20:5-5,8,11,14,17 cis**	4.187±0.034
**C22:1-11 cis**	2.220±0.025
**C22:6 n-3**	10.183±0.146

Fatty acid methyl esters (FAME) were prepared by transesterification with KOH solution in methanol (c = 2 mol/L) according to the ISO 5509:2000 method [69] and analyzed by an Agilent Technologies 6890N Network GC System gas chromatograph (Santa Clara, CA, USA) with flame ionization detector.

69. ISO 5509:2000; Animal and Vegetable Fats and Oils—Preparation of Methyl Esters of Fatty Acids. ISO: Geneva, Switzerland, 2000.
